# Epidural Catheterization in Obstetrics: A Checklist-Based Video Assessment of Free Available Video Material

**DOI:** 10.3390/jcm11061726

**Published:** 2022-03-20

**Authors:** Armin N. Flinspach, Florian J. Raimann, Richard Schalk, Lena Bepler, Miriam Ruesseler, Mairen H. Flinspach, Kai Zacharowski, Jasmina Sterz

**Affiliations:** 1 Department of Anaesthesiology, Intensive Care Medicine and Pain Therapy, University Hospital Frankfurt, Goethe University Frankfurt, 60590 Frankfurt, Germany; florian.raimann@kgu.de (F.J.R.); richardschalk71@googlemail.com (R.S.); kai.zacharowski@kgu.de (K.Z.); 2 Frankfurt Interdisciplinary Simulation Center FIneST, Medical Faculty, University Hospital Frankfurt, Goethe University Frankfurt, 60590 Frankfurt, Germany; lena.bepler@kgu.de (L.B.); miriam.ruesseler@kgu.de (M.R.); jasmina.sterz@kgu.de (J.S.); 3 Department of Trauma, Hand and Reconstructive Surgery, University Hospital Frankfurt, Goethe University Frankfurt, 60590 Frankfurt, Germany; 4 Department of Anaesthesiology, Intensive Care Medicine and Pain Therapy, Sana Clinic Offenbach GmbH, 63069 Offenbach am Main, Germany; mairen.flinspach@sana.de

**Keywords:** obstetrics, obstetric pain, pain management, instructional film and video, anesthesia, epidural, education

## Abstract

Epidural catheterization has become an indispensable part of modern pain therapy, for example, in obstetrics. Learning how to master this skill is an important competency. Videos are among the information sources with the highest information content for learning such skills. The present study aims to analyze videos regarding epidural catheter placement provided on the YouTube platform based on a validated checklist. An expert workshop selected crucial items for learning epidural catheterization in obstetrics. Items were identified and optimized in a five-step testing process. Using this checklist, videos from YouTube were evaluated by eleven health care professionals. Sixteen videos were identified and analyzed. Concerning the catheterization-specific part of the checklist, only two videos showed satisfactory quality. In the didactic part, eleven out of 21 items reached a mean score >50% of the points. Regarding interrater reliability, the catheterization-specific checklist was shown to be substantial (Fleiss’ kappa = 0.610), and the didactic part was shown to be fair (Fleiss’ kappa = 0.401). Overall, standard monitoring and appropriate aseptic technique were followed in only 42% and 49% for the procedure. There was a significant correlation between the runtime and the content quality (*p* < 0.001). No correlation could be found in terms of platform rating parameters. The video quality varied highly in terms of the requirements of this practical skill. The majority appear unsuitable for self-study due to serious errors and deficiencies regarding patient safety. However, there is no quality control on free platforms. Accordingly, it is difficult to identify suitable videos for educational purposes.

## 1. Introduction

Epidural catheterization is a safe and frequently used procedure in modern anesthesia and has become an integral part of modern obstetrics in general [1]. Therefore, the performance of an epidural puncture with appropriate catheter placement is a high-priority skill in the anesthesiology education curriculum [2,3].

Currently, obtaining information from the internet is a part of everyday life for medical students, trainees, professionals and nonprofessionals, especially due to the benefits of video learning [2]. One of the most informative sources of information for learning practical skills is videos. Medical students themselves predict that they can learn more effectively with videos than with books, plain texts, or images [4]. Thus, they prefer educational videos, especially for learning procedures such as practical skills [5,6]. This becomes even more important as it has been claimed that many members of Generation Z (students born after 1995) see themselves as “observers” and want to watch others complete tasks before applying the learning themselves. Therefore, they use videos that are available free of charge on the largest portal of this kind: YouTube [7].

However, it is not easy for the users of these portals to determine the professionalism and correctness of intervention or the didactic quality of the videos by the existing rating system or the number of clicks [8]. Thus, to illustrate to physicians the quality of the content they consume for further training or instruction purposes, as well as to enable laypersons to gain professional insight into the subject matter, it is necessary to label the videos according to uniform, reproducible standards.

For the qualitative evaluation of educational video material, Rüsseler et al. designed and evaluated a didactic questionnaire to assess the quality of videos [9]. However, there has not yet been an investigation into the application of epidural catheter placement relevant to anesthesia and pain therapy.

Therefore, the present study aims to analyze videos regarding epidural catheter placement provided on YouTube based on a validated checklist. Furthermore, we analyzed whether the parameters provided by YouTube-like ratings or likes correlated with the quality of these videos.

## 2. Methods

### 2.1. Ethical Approval

The study was conducted according to ethical principles of the Helsinki Declaration (Ethical Principles for Medical Research Involving Human Subjects). Since the present study only involved an evaluation of publicly available videos and was not considered clinical research on human subjects, no ethics vote was requisite according to the guidelines of the ethics committee of the University Hospital of Frankfurt. 

### 2.2. Elaboration of the Checklist Items

Initially, an expert panel with four experienced anesthesiologists working at different academic hospitals, certified according to German medical specialist standards, with a high level of obstetric experience was set up. These experts were asked to define the crucial content for learning how to perform an epidural puncture and epidural catheterization in obstetrics. The drafting was carried out in strict accordance with the international guidelines of the American Society of Anesthesiologists Task Force on Obstetric Anesthesia and the Society for Obstetric Anesthesia and Perinatology, Obstetric Anaesthetists’ Association (OAA) and national guidelines [10,11,12].

The subsequent consensus process using a three-stage modified Delphi method guided by experts in medical education (master’s degree in medical education) [13].

This led to the identification of 20 items, which were clustered into 4 main sections (initial procedure, sterile work, puncture, further procedure) and drafted into a checklist.

### 2.3. Optimization and Analysis of the Checklist

The checklist evaluation was conducted in a multi-step process and included in particular [14]:

1. Validity:

The items were repeatedly reviewed for consistency with currently available guidelines for epidural catheterization [10,11,12].

2. Reliability:

The averaged correlation between items within repeated test applications of the checklist.

Test-retest reliability: A subset of reviewers was asked to repeat the checklist a second time a few days later, and the correlation between measurements was assessed.

3. Acceptability and feasibility:

Measured by the successful completion rate and time taken to complete the checklist.

For this purpose, experienced anesthesiologists rated videos dealing with an epidural puncture and epidural catheterization in obstetrics. This rating was performed without prior training or the possibility of peer agreement. Afterwards, the individual ratings were analyzed and critically discussed by the reviewers and the authors, particularly regarding the reasons for the points awarded, strengths, weaknesses, and individual meaning of the items. Based on this, the items were adapted and specified to achieve a common and mutual understanding for rating each item. Finalization was achieved by checklist retesting. The resulting checklist can be found in Appendix A.1.

The skill-specific checklist was complemented by the validated didactic and audiovisual video quality checklist published by Rüsseler et al. [9].

### 2.4. Collection and Evaluation of the Videos

For video evaluation, data were obtained based on the corresponding video search in February 2017 on the YouTube portal (www.youtube.com) in both German and English using the search terms “epidural” and “peridural.” The data were collected individually and in combination with the keywords: “birth”, “obstetrics”, “delivery”, “catheter”, “anesthesia” and “anaesthesia”.

Over 600 videos were examined by independent investigators regarding suitability to demonstrate epidural anesthesia (for detailed inclusion and exclusion criteria, see Table 1). A total of 16 videos were acquired for study purposes (last video access made on 5 September 2021). For each video, the number of views, likes, dislikes, uploaders/producers, date of upload, runtime, and channel subscribers were recorded for analysis purposes. In addition, the videos were assigned a category according to the content creator. In this context, the investigators achieved consensus on the classification of content creators into one of three groups: medical societies, hospitals or hospital chains, and others.

The primary criterion for suitability as an educational resource was defined a priori as percentage achievement levels. Videos with an achievement ≥80% of the maximum number of points were considered to be recommendable, a predominant suitability was defined at ≥60%, and achievement of at least 50% was assumed to be at partial suitability [15].

### 2.5. Data Collection

For the video evaluation, each video was rated by four anesthesiologic senior physicians, two anesthesia care specialists, three undergraduate medical students, and two experts in medical education (a total of eleven raters per video) who rated the videos using the checklist. The rating was performed without prior training or the possibility of mutual agreement. In addition, the age, profession, and clinical experience of the raters were recorded. The video assessment results were collected using pen and paper-based checklists implemented in Excel (Windows Excel, Microsoft^©^, Redmond, WA, USA) for further data preparation.

### 2.6. Statistical Analyses

Data were collected using Excel. We predefined a statistical analysis plan prior to the study. Data analysis was performed using SPSS (IBM Corp., Version 26, Chicago, IL, USA). Data with continuous scales are represented as the mean (± standard deviation), and data with categorical scales are presented as frequencies and percentages. Interrater reliability was analyzed using Fleiss’ Kappa. Correlations were examined with a Spearman’s rho test. A *p*-value of 0.05 or less was considered statistically significant.

## 3. Results

Overall, over 600 videos were found using the defined search terms. Of these, only 16 withstood a primary examination concerning the exclusion criteria (see Table 1). The average length of the videos was 7:20 min, the median number of views was 42,258 (range: 491–612,320 views), and the videos received a median of 64 likes. In terms of content creators, we were able to assign two videos to professional societies, eight videos were created by hospital networks, and six videos were produced solely by a medical professional. On average, the videos achieved 48.7% of the possible points, with 10 videos not reaching the threshold of 50% of the checklist requirements.

In the catheterization skills-specific part of the checklist, one video was rated with a median >80% (best video = 80.9%), and another video had a median >60% (second-best video = 69.1%). The six top-rated videos are shown in Table 2.

In this part of the checklist, only six of the 20 items defined in the checklist were evaluated as correct in more than 50% of the videos. These items were local anesthesia (98.3%), sterile wash down (60.8%), sterile gloves (55.1%), proper positioning (54.0%), catheter insertion (53.4%), and wound dressing (52.0%). The items most unlikely rated as performed correctly were emergency equipment available (5.1%), hand disinfection (9.7%), and aspiration tests (13.1%). The completeness of the questionnaires in the skills-specific section was 38.6% (±17.0%), and in the didactic section, it was 52.6% (±15.4%). In the didactics section, the mean rating of the videos was 52.6%. Here, a mean score >50% was achieved in eleven of 21 items, and one video reached 93.2% of the maximum score. 

Moreover, four videos achieved a median of >60%. The six items with the highest ratings were visual quality (83.4%), audio quality (78.9%), sequence (74.7%), appropriate title (70.0%), target audience (64.4%), and scientific accuracy (63.0%). The lowest rated didactic items were content summary (11.4%), giving in-depth literature (25.1%), and judicious use of insertions (34.5%).

The interrater reliability (IRR) could be determined as substantial (Fleiss’ kappa = 0.610) for the catheterization skills-specific part of the checklist and as fair (Fleiss’ kappa = 0.401) for the didactics part of the checklist, resulting in an overall IRR of 0.542 (moderate) [16]. In particular, the catheterization skills-specific section showed a high IRR for the individual test items. In the didactic section, a moderate IRR could only be demonstrated for three modules (readability, duration of insertion, and quality of text/graphics). A detailed evaluation is shown in Table 3.

Concerning the content ranking parameters reported by YouTube for the videos, there was a significant correlation between the runtime and the content quality (*p* < 0.001). In terms of likes (*p* = 0.88), dislikes (*p* = 0.87), number of views (*p* = 0.51), or subscribers (*p* = 1.00), no correlation could be found. This is also evident from the scores of the best videos shown in Table 2 (for example, video six with only 35.9% content completion).

The top-rated video, which was uploaded by a recognized German medical society and published by a group of authors in the New England Journal of Medicine, received 89.8% of all points but had only 491 views and five likes.

With respect to the scientific background classification of the content creators, a significant correlation was found with a higher average score (*p* < 0.01).

## 4. Discussion

In the present study, the quality of YouTube videos regarding epidural catheter placement was analyzed using a validated checklist. We were able to identify over 600 videos on YouTube based on our search terms. However, in the present study, only 16 videos met the inclusion criteria. Furthermore, we were able to show that only one video met the content requirements for the practical skill of epidural catheterization. This result is even more relevant considering the high relevance of this practical skill to the field of anesthesiology. Epidural catheter placement is one of the most important procedures in the field of acute pain therapy, both in the intra- and postoperative setting after major abdominal or thoracic surgery, as well as in obstetrics.

Of the 16 included videos, only one scored higher than 80% on the checklist. These findings are concordant with the results published in various studies that analyzed educational videos regarding other medical skills with respect to both the low number (e.g., Fischer et al. were only able to evaluate 13 videos on knee puncture) and the low educational quality of the videos [8,17,18,19].

In the present work, we observed a lack of adherence to the aseptic performance of puncture and catheter placement. While the absence of essential hand disinfection can perhaps still be subsumed as a self-evident step, this is no longer possible concerning the need to wear sterile gloves or keep the field sterile. This is simply inadequate if not present. Even if the number of epidural infections after puncture and catheter placement is low, a strictly aseptic procedure must also be strictly adhered to [20,21,22]. However, compared to other skills to be performed under sterile conditions, we showed far lower fulfillment of the requirements. For example, Fisher et al. analyzed the quality of videos regarding knee arthrocentesis provided by YouTube. For this procedure, which has similarly high demands on sterility as epidural catheter placement, the authors were able to show that only 46% of the videos showed correct sterile conditions [17].

In addition, it is noticeable that the majority of videos inadequately depict the aspect of patient monitoring concerning vital monitoring (42.0%) and the availability of emergency equipment (5.1%). This is even more remarkable, as epidural puncture and catheterization can cause various potentially serious and even life-threatening complications (e.g., accidental, unrecognized spinal anesthesia) and, in the case of deliveries, is predominantly not performed at a primary anesthesia workstation with the correspondingly self-evident provision [1,23]. In particular, to reduce serious complications such as epidural hematoma and undetected accidental misplacements, the low number of completely fulfilled items regarding aspiration tests (*n* = 2; 12.5%) and catheter insertion (*n* = 5; 37.5%) was surprising.

Until now, there has been no internationally concerted checklist of quality requirements to which professional societies, commercial or private content creators could be guided. The New England Journal of Medicine is doing valuable pioneering work in this regard with peer-reviewed videos in clinical medicine [24].

Checklists, such as the one we have drafted, should form the basis for instructional videos on a practical skill to ensure sufficient video quality. This is foreseeably not realistic for YouTube, despite the fact that this platform will remain one of the main sources due to fast app-based availability and media reach. In this respect, it can only be recommended to use only sources that produce verified, reliable and accurate information. The strength of the checklist developed in the present study lies in the concerted quality of the content and the high interprofessional IRR.

A distinctive aspect of the epidural catheter during childbirth is the high public awareness. This also frequently generates a request for further information, which tempts pregnant women and learners to search for relevant information on video platforms such as YouTube. According to our results, an objective evaluation and validation of video material is possible with simple methods and should be conducted to avoid inadequate information transfer, false expectations, concerns, or incorrect performance. The fact that the criteria provided by YouTube for evaluation, such as views or likes, showed no correlation with content quality in our study or previous studies is particularly unfortunate [25,26]. This becomes even more relevant in the awareness that the sequence of the hundreds to thousands of videos available is determined by the algorithms of the YouTube platform, which are guided by likes and views. However, it must also be noted that the content available on the YouTube video platform is subject to very dynamic processes. Among other things, this is due to the uncomplicated copying and editing of content and the frequent presence of various sub forms of a video the authors deliberately decided to exclude duplicates. However, the number of views, as well as likes and channel subscriptions, are also subject to a highly dynamic situation. Any comparison can only be based on a specific point in time. Further analyses such as the increase in likes or subscriptions over time are unfortunately limited. Nevertheless, the urgent need for appropriate evaluability using reliable checklists, such as the one we developed for epidural catheter placement, remains. While content may change within seconds, adequate tools, such as checklists, for assessing such video material remain rare and may be applied to new content at any time in the future.

In the present study, some limitations should be taken into account. One of these is that only videos from the YouTube platform were analyzed. However, since this is the largest and most frequently used platform, the analysis, in particular, appears to be appropriate [27]. When evaluating the checklist items, the individual contents were not prioritized with regard to their particular importance, as an objectifiable classification was not possible. Based on the complex data evaluation, the search for suitable video material is subject to a time lag before publication, which is not insignificant. Due to the COVID-19 pandemic and the associated restrictions on classroom teaching at universities, lecturers were increasingly faced with the challenge of making web-based educational sources available. It can therefore be assumed that the number of videos available on YouTube regarding epidural catheter placement has increased again. However, the algorithms of the YouTube platform have changed as well, so a direct comparison is inappropriate. Further investigations should address the question of an improvement in content due to the more recent broadness of video availability.

In addition, we examined videos that are suitable for demonstrating practical medical skills but without assigning the primary recipient. For the skill of a labor epidural analgesia catheter placement, this can indisputably also be a patient or a pregnant woman. Since the primary recipient is usually not mentioned in the videos, it must be assumed that medical students or young doctors, in particular, will view and use the videos regardless of the primary recipient.

Since only one of the videos examined appears to be recommendable, it would be desirable to carefully create new videos based on the tested checklist. Thus, the correct and clear presentation of the necessary interventions could already be ensured during video creation.

## 5. Conclusions

The quality of the videos on ‘YouTube’ showing how to perform epidural punctures varies widely. The majority of videos have serious errors and deficiencies, highly endangering patient safety. Even though the target group of the videos was not exclusively medical learners, the viewing was performed by them. Accordingly, these videos should predominantly be described as unsuitable for self-study.

However, there is no transparent quality control on free video platforms such as YouTube. A concerted quality seal for instructive, accurate videos, if possible, would also be useful on public platforms. Regarding the practical skill of epidural puncture and positioning of a catheter, we would hope to contribute to such a tool in the present work. The quality of our items seems adequate in respect of the high degree of agreement among different medical target groups.

## Figures and Tables

**Table 1:** Video Search: Inclusion and Exclusion criteria.

Inclusion Criteria:		
Video search terms: combinations with “epidural” or “peridural”		
and:	“anaesthesia” and “birth”	respectively “anesthesia”
	“anaesthesia” and “obstetrics”	respectively “anesthesia”
	“anaesthesia” and “labour”	respectively “anesthesia”
	“catheter” and “birth”	
	“catheter” and “obstetrics”	
	“catheter” and “labour”	
Exclusion criteria:		
	Duplicate videos *	
	Videos in languages other than English or German	
	Videos without sound	
	Videos without picture	
	Birth reports	
	sole animations	

Report of the applied search terms in order of inclusion criteria, as well as exclusion criteria for the selection of suitable video material on the YouTube platform for evaluation regarding epidural catheter placement. * In the case of duplication, the video with the higher reach in terms of views and likes was included in the evaluation.

**Table 2:** Top rated videos.

Video	URL	Runtime *^a^*	Views	Likes	Dislikes	A-Section	B-Section
1	https:/youtube.com/watch?v=VBtgseSpPMc	14:29	491	5	0	80.9%	93.2%
2	https:/youtube.com/watch?v=CGhjMYSr18M	12:47	117,233	112	43	69.1%	69.8%
3	https:/youtube.com/watch?v=8SRQmLkIG7s	05:45	6294	12	4	48.0%	49.5%
4	https:/youtube.com/watch?v=wbti9kKcuEw	07:23	2825	10	2	45.0%	64.8%
5	https:/youtube.com/watch?v=_URgMM4yTIQ	09:49	14,026	63	7	42.7%	58.3%
6	https://youtube.com/watch?v=9_y8gnZZDaQ	11:52	612,320	737	292	35.9%	60.9%

Top rated videos: Top six rated epidural catheterization videos with associated rating information from the YouTube platform and results of the checklist-based review. Video last accessed 5 September 2021; *^a^* Runtime presented in minutes: seconds. Abbreviations: A-Section, epidural catheterization specific items; B-Section, Video-didactic specific items; URL, uniform resource locator.

**Table 3:** Consistency of ratings by reviewers for skills specific items: Interrater reliability.

Checklist Item	IRR *^a^* (Fleiss’ Kappa)
Initial Measures	
appropriate peridural needle	0.463
Vital monitoring (ECG, BP)	0.401
Carrying out CTG monitoring	0.754
Emergency equipment available	0.602
Presence of an assistant	0.503
Sterile working	
hygienic hand disinfection	0.805
sterile gloves	0.582
Wearing hair net, etc.	0.632
Sterile cleaning	0.268
Puncture	
Correct positioning	0.435
Puncture site selection	0.483
Local anesthesia	1.000
Needle guidance:	0.475
Loss of Resistance Technique	0.589
Catheter insertion	0.384
Catheter depth	0.669
Further procedure	
Filter usage	0.602
Aspiration test	0.701
Wound dressing	0.523
Connection monitoring	0.349

Interrater reliability of the catherization specific part of the checklist. ***^a^*** Interpretation of the interrater reliability (Fleiss’ Kappa): <0.2 slight, 0.21–0.40 fair, 0.41–0.60 moderate, 0.61–0.8 substantial, 0.81–1.0 perfect, [11]; Abbreviation: IRR, Interrater reliability; ECG, electrocardiography; BP, blood pressure; CTG, cardiotocography.

## Data Availability

Raw data were generated at University Hospital of Frankfurt. Derived data supporting the findings of this study are available from the corresponding author ANF on reasonable request.

## Appendix A

### Appendix A.1. Skill Specific Checklist Section for Epidural Catheter Positioning

**Task**	**Not Mentioned** **(0 P.)**	**Incorrect** **/Incomplete** **(1 P.)**	**Correct (2 P.)**	**Not Applicable** **/Already Done**
Initial measures				
Appropriate peridural needle	☐	☐	☐	-
2p naming a needle (e.g., Tuohy)				
Vital monitoring (ECG, BP)	☐	☐	☒	-
2p visible established				
Carrying out CTG monitoring	☐	-	☐	-
Emergency equipment available	☐	-	☐	-
Presence of an assistant	☐	☐	☐	-
A midwife and/or an anesthesia nurse				
1p uninvolved 2p active assistance				
Sterile working				
hygienic hand disinfection	☐	-	☐	-
sterile gloves	☐	-	☐	☐
Wearing hair net, mouth guard, sterile coat	☐	☐	☐	☐
Sterile cleaning	☐	☐	☐	-
only 1p in case of contamination in the course				
Puncture				
Correct positioning	☐	☐	☐	-
sitting: cat hump, shoulders hanging lying: embryonic position				
2p Demonstration of performance				
Puncture site selection	☐	☐	☐	-
Median puncture Th10-L2				
2p detailed explanation				
Local anesthesia	☐	-	☐	☐
Needle guidance	☐	☐	☐	-
Stabilization to the patient, insertion with stylet				
Adjusting the puncture angle				
Loss of Resistance Technique	☐	☐	☐	-
2p explain and demonstrate				
Catheter insertion incl. needle removal	☐	☐	☐	-
Catheter depth (≤5 cm above LOR)	☐	☐	☐	-
2p incl. explanation of depth determination				
Further procedure				
Filter usage	☐	-	☐	-
Aspiration test	☐	☐	☐	-
2p check for blood and CSF				
Wound dressing	☐	-	☐	-
Connection monitoring	☐	-	☐	-
2p Already at nomination				
Translation (original in German) of the developed and applied specific checklist for the evaluation of the correctness of epidural catheter positioning. Abbreviations: BP, non-invasive blood pressure; EKG, electrocardiography; CSF, cerebrospinal fluid.			

### Appendix A.2. List of All You-Tube Video Search Terms: Inclusion and Exclusion Criteria

Inclusion criteria:

The first 50 video results on the Youtube platform for the following search terms were taken into account. All combinations with “epidural” or “peridural” and:

anaesthesia and birth	respectively anesthesia and birth
anaesthesia and obstetrics	respectively anesthesia and obstetrics
anaesthesia and labour	respectively anesthesia and labour
catheter and birth	
catheter and obstetrics	
catheter and labour	

Video exclusion criteria:

- Duplicate videos
- Videos in languages other than English or German
- Videos without sound
- Videos without picture
- Birth reports
- Sole animations
